# Novel Biomarkers for Rejection in Kidney Transplantation: A Comprehensive Review

**DOI:** 10.3390/jcm14155489

**Published:** 2025-08-04

**Authors:** Michael Strader, Sam Kant

**Affiliations:** 1 School of Medicine, University College Dublin, Belfield, D04 V1W8 Dublin, Ireland; drmichaelstrader@gmail.com; 2 St. Vincent’s University Hospital, D04 T6F4 Dublin, Ireland

**Keywords:** transplant, biomarkers, rejection, immunosuppression

## Abstract

Kidney transplantation is the treatment of choice for patients with end-stage kidney disease. Despite significant advances in graft survival, rejection continues to pose a major clinical challenge. Conventional monitoring tools, such as serum creatinine, donor-specific antibodies, and proteinuria, lack sensitivity and specificity for early detection of graft injury. Moreover, while biopsy remains the current gold standard for diagnosing rejection, it is prone to confounders, invasive, and associated with procedural risks. However, non-invasive novel biomarkers have emerged as promising alternatives for earlier rejection detection and improved immunosuppression management. This review focuses on the leading candidate biomarkers currently under clinical investigation, with an emphasis on their diagnostic performance, prognostic value, and potential to support personalised immunosuppressive strategies in kidney transplantation.

## 1. Introduction

Kidney transplantation remains the gold standard treatment for patients with end-stage kidney disease requiring kidney replacement therapy [1]. Over several decades there has been improvement in kidney graft survival and patient survival, despite increasing patient comorbidities and an older deceased donor pool. This has been due to an improved understanding of the innate immune system, improved therapeutic immunosuppression, and access to immunosuppression. However, despite these advances, the risk of graft failure is approximately 12% within 1 year and 10–20% within 5 years due to T-cell mediated rejection (TCMR) and antibody mediated rejection (ABMR) [1,2,3]. The broadened understanding of the pathophysiology of transplant rejection has highlighted the current limitations in clinical diagnosis, most notably, our continued reliance on the presence of donor-specific antibodies (DSA) as markers of rejection [4,5]. Fortunately, newer non-invasive biomarkers and molecular diagnostic tests have come into focus for earlier detection of rejection and accurately phenotyping rejection.

As in other domains of nephrology, non-invasive biomarkers are increasingly sought after due to their ability to provide rapid, accessible, and lower-risk alternatives to biopsy. However, the Banff biopsy criteria remain the current “gold standard” for the diagnosis of rejection, despite known limitations, such as inter-observer variability and procedural risks from biopsy [6,7,8,9]. Serum creatinine and proteinuria, though routinely used, are late and non-specific markers of graft dysfunction. Moreover, transplanted patients often experience multiple concurrent complications, such as immunosuppression-related nephrotoxicity, infections, malignancies, weight loss, hypertension, and new-onset diabetes, which can further obscure the clinical picture and complicate the differentiation of rejection from other causes of graft injury in the absence of a biopsy. As such, long-term transplant management remains challenged by the lack of sensitive and specific non-invasive tools [1,4,5].

Recent advances in biopsy molecular phenotyping, demonstrated by the Molecular Microscopic Diagnosis System (MMDx) project has led to phenotypic enrichment and has uncovered the limitations of conventional histology [10,11,12]. MMDx incorporates a ‘nearest neighbour’ analysis, comparing the transcriptomic profile of the patient’s biopsy with those in a reference database of fully phenotyped samples. This multivariate approach assesses similarity to known outcomes, enabling both diagnostic classification and risk stratification. MMDx leverages the strength of ensemble machine learning methods and has uncovered discordances between histology and molecular signals, thus highlighting conventional histological limitations [10,13,14,15,16]. However, although MMDx continues to improve histological molecular diagnosis, it is limited by the necessity of a biopsy. Therefore, non-invasive biomarkers obtained from blood and urine are needed to reduce cost and graft risk from invasive biopsies.

A recent review highlighted novel biomarkers in immune mediated rejection that are close to market and translation into clinical practice with commercially available assays [17]. In this review, we report promising novel biomarkers close to clinical translation, both blood and urine based, that are undergoing clinical trials in the field of kidney transplant rejection.

## 2. Donor Derived Cell Free DNA

### 2.1. Background

Donor-derived cell-free DNA (dd-cfDNA) refers to fragmented DNA released into the bloodstream or urine because of cell apoptosis or necrosis. It has garnered increasing interest as a non-invasive “liquid biopsy” for detecting acute, chronic, and subclinical rejection. Its clinical utility is supported by growing evidence demonstrating its diagnostic accuracy in identifying graft injury and rejection episodes [18,19,20,21]. Recent advances in assay technology have facilitated its integration into clinical practice, with several tests now commercially available [17].

### 2.2. Clinical Utility

In stable patients, dd-cfDNA was demonstrated to be low, thus supporting its role in active rejection and reassurance in graft stability [19]. In contrast, dd-cfDNA levels rise significantly during episodes of rejection. The Circulating Donor-Derived Cell-Free DNA in Blood for Diagnosing Acute Rejection in Kidney Transplant Recipients (DART) study, the first multicentre study of renal allograft recipients using a validated dd-cfDNA test, employed targeted amplification and sequencing of single-nucleotide polymorphisms to quantify donor and recipient DNA contributions without prior genotyping. It validated a threshold of ≥1% dd-cfDNA fraction, defined as the proportion of dd-cfDNA relative to total cell-free DNA, demonstrating high diagnostic discrimination and a high negative predictive value for ruling out rejection [20].

Recently, a study by Aubert et al. [21] examined the relationship between dd-cfDNA levels and histological findings on biopsy. Their study showed that dd-cfDNA not only correlated with both the presence and severity of graft injury but also enhanced the predictive power of standard clinical models. Furthermore, the Trifecta Working Group conducted an international prospective study to explore how dd-cfDNA levels measured at the time of indication biopsy correlate with underlying molecular signals and histological diagnosis. They demonstrated that dd-cfDNA can be elevated in cases of histologically confirmed rejection even in the absence of detectable DSA, reinforcing its sensitivity as a biomarker of alloimmune injury [15,22].

To enhance diagnostic accuracy, some studies have proposed combining the dd-cfDNA fraction with its absolute concentration (copies/mL) [15,23]. Notably, the combination of the two improved accuracy and re-classified missed rejections [15]. Additionally, the findings supported the correlation of dd-cfDNA with molecular diagnostics, measured through MMDx [13,14,15].

Multiple systematic reviews have been conducted to report the clinical validity of dd-cfDNA and highlight methodological limitations [24,25]. In the most recent meta-analysis, which included 22 papers, it demonstrated that dd-cfDNA had a pooled AUC of 0.80 (95% CI, 0.76–0.83) and 0.83 (95% CI, 0.76–0.88) negative predictive value for any type of rejection. They also demonstrated increased accuracy to ABMR [26]. These findings reinforce the clinical value of dd-cfDNA as a sensitive, non-invasive tool for detecting allograft rejection.

Although many studies have looked at dd-cfDNA as a diagnostic marker, it has also been evaluated as a prognostic marker. Several studies have demonstrated that variability, or increased levels, of dd-cfDNA were associated with rejection and graft function decline [27,28]. Additionally, dd-cfDNA may help determine treatment response after modification of immunosuppression [29,30].

Collectively, these studies demonstrate that dd-cfDNA is a clinically validated, non-invasive biomarker that reflects active alloimmune injury. It correlates with both histological and molecular markers of rejection and offers prognostic insight into graft outcomes. Its sensitivity in the absence of DSA, ability to enhance standard models, and potential to monitor treatment response highlight dd-cfDNA’s expanding role across the continuum of transplant care.

### 2.3. Limitations of dd-cfDNA

Although dd-cfDNA is a promising non-invasive biomarker for allograft rejection, there remains hesitancy around its use as a standalone diagnostic tool. Its lack of specificity, being elevated in other forms of allograft injury, such as infection or biopsy-related trauma, necessitates interpretation with clinical context [31]. Furthermore, dd-cfDNA has limited sensitivity in detecting chronic pathological changes such as interstitial fibrosis and tubular atrophy, and its levels can be confounded by post-transplant kinetics or fluctuations in recipient cfDNA levels [32,33]. Methodological issues, including potential underestimation with polymerase chain reaction (PCR)-based assays, also pose challenges [1,17]. As such, dd-cfDNA may be best interpreted alongside complementary biomarkers or clinical data to improve specificity and better characterise the underlying cause of graft injury.

### 2.4. Future Directions of dd-cfDNA

It may be the combination of dd-cfDNA with traditional histopathology that provides the most clinical value. In context, dd-cfDNA may indicate the need for a biopsy, or it can be used with the biopsy results to correctly categorise rejection [34]. This was highlighted by the Banff Minimally Invasive Biomarkers Working Group, where they discussed the potential of dd-cfDNA when integrated into existing Banff histological criteria [35]. Large cohort studies are ongoing (e.g., NCT04239703, NCT06013358, NCT06025240) which may help further elucidate dd-cfDNA’s role in transplant rejection [21,36]. The results of these trials aim to clarify the biomarker’s utility across diverse transplant settings and help define its role in transplant monitoring.

## 3. Chemokines

### 3.1. Background

Urine is a direct reflection of transplanted graft function; therefore, a urine biomarker would be an ideal non-invasive surrogate measurement of ongoing function and injury. The C-X-C motif ligands (CXCL) 9 and 10, which are CXC chemokine receptor-3 binding ligands, are quantified within the urine and are the most studied and validated chemokines in the setting of transplant rejection [37,38,39,40,41]. They regulate leukocyte trafficking and migration during the inflammatory response associated with TCMR and ABMR [38,42,43]. Low levels of CXCL may indicate immunological quiescence and therefore prognosticate risk of rejection [44].

### 3.2. Clinical Utility of Chemokines

Similar to dd-cfDNA, chemokines are potential candidate biomarkers due to their non-invasive nature of collection and association with immune TCMR and ABMR; both CXCL9 and CXCL10 have been investigated, both creatinine-corrected and non-corrected, with both having high negative predictive values and being highly associated with acute rejection [41]. The Clinical Trials in Organ Transplantation Protocol (CTOT-1 and CTOT-4) studies, which were multicentred prospective observational trials to validate the diagnostic and predictive ability of novel candidate biomarkers for transplant outcomes, demonstrated that CXCL levels rise before acute rejection is clinically determined, therefore providing prognostic enrichment [41,45].

A study conducted by Tinel et al. [37] sought to investigate potential confounders and create a model for the clinical application of CXCL9 and CXCL10. They noted that CXCL levels were raised when DSA were positive and upregulated in BK virus infections (both viremia and nephropathy). The clinical model incorporated eight variables across four domains—clinical factors (recipient age and sex), biological markers (estimated glomerular filtration rate (eGFR) and DSA score), potential confounders (BK virus viremia and urinary tract infection), and urinary chemokines (CXCL9/Cr and CXCL10/Cr)—and significantly enhanced the ability to predict acute rejection risk (AUC 0.85; *p* < 0.0001) and correctly re-classify patients. Interestingly, the model also performed best with the addition of both CXCL9 and CXCL10, thus supporting the idea that both markers independently provide granularity in the context of rejection.

In comparison, a subsequent study by Van Loon et al. [46] (NCT02832661) reported moderate diagnostic performance of creatinine-corrected urinary CXCL9 and CXCL10 for acute rejection, with AUCs of 0.72 (95% CI, 67.3–76.7) and 0.70 (95% CI, 65.5–75.1), respectively. However, diagnostic accuracy improved when limited to patients who received anti-rejection therapy (AUC 0.80 for CXCL9/Cr; 0.76 for CXCL10/Cr) and was higher in indication biopsies and cases of ABMR versus TCMR. Notably, in a multivariable mixed model, five variables were independently associated with rejection: higher log-transformed CXCL9 and CXCL10, lower eGFR, presence of Human Leukocyte Antigen-DSA, and lower BK viremia. Additionally, both chemokines showed a dose–response relationship with inflammatory severity and were prognostic of graft loss when stratified by quartiles.

In contrast to other chemokine studies, the KTD-Innov study (NCT03582436), which was a large prospective trial aimed at investigating CXCL9 and CXCL10 as rejection biomarkers, found that the chemokines were associated with rejection, but only CXCL9 had minimal clinical benefit over standard of care parameters. Therefore, the authors concluded that the addition of the chemokines would be unlikely to guide clinical decisions [47]. However, these conflicting results could be due to a low incidence in the primary outcome, use of different assays, and use of a single population. Therefore, although many studies suggest that urinary CXCL9 and CXCL10 hold promise as non-invasive biomarkers for early detection and risk stratification of acute rejection, further large international multicentred prospective trials will be needed to determine the clinical utility of the CXCL biomarkers in transplant rejection.

### 3.3. Limitations

Due to the immunological nature of the CXCL proteins, they are often up-regulated in settings of non-rejection, such as infections, acute kidney injury, and autoimmune diseases of the kidney [40,46,48,49]. Therefore, their specificity for rejection is limited. However, present clinical tools can often rule out these confounders, and as a non-invasive tool with a high negative predictive value it may provide clinical guidance and screening for rejection [37].

### 3.4. Future Directions of Chemokines

With two recent large cohort trials demonstrating potential clinical models for application, further trials are required to validate their application. The KTD-Innov study, a large prospective trial, reported findings that differed from earlier studies, thereby raising questions about the clinical applicability of chemokines in transplant rejection [47]. Therefore, the ongoing clinical trials (NCT03206801, NCT06564649, NCT06351488, and NCT03719339) may provide further granularity into the application of the chemokines and expand on existing models for potential clinical translation.

However, variability in performance across trials, as highlighted by the limited added value reported in KTD-Innov, underscores the influence of study design, assay standardisation, and patient selection. As such, while CXCL chemokines consistently demonstrate immunological relevance and prognostic value, their role as a standalone diagnostic tool, like dd-cfDNA, remains uncertain. Future evidence is likely to position them within integrated, multi-biomarker strategies, where their contribution may be optimised in synergy with other non-invasive markers such as dd-cfDNA or mRNA signatures.

## 4. Torque Teno Virus

### 4.1. Background

The presence of BK viremia often reveals a net state of immunosuppression. Recently, Torque Teno Virus (TTV), a non-enveloped deoxyribonucleic acid (DNA) virus not associated with pathological diseases, has come to the forefront as a candidate biomarker in transplantation. TTV levels are inversely proportional to the number of active T lymphocytes, are unaffected by conventional antiviral therapies, and therefore serve as a useful indicator of functional immune competence [50,51,52]. As such, TTV shows promise as a biomarker for assessing a patient’s immunosuppressive state, potentially guiding immunosuppressive therapy to minimise the risks of both over- and under-immunosuppression, and enabling more personalised transplant management. A recent review article and meta-analysis on TTV describe current application potential and limitations amongst the literature [53,54].

### 4.2. Clinical Utility TTV

In 2017, the Vienna group reported that higher TTV levels were associated with a reduced risk of ABMR, with a relative risk ratio of 0.94 per TTV log (95% Confidence Interval (0.90–0.99)). Interestingly, they also noted differences in TTV viral load based on the types of immunotherapies used, such as tacrolimus versus mTOR inhibitors, and induction therapy [55]. Subsequent analyses from the same group showed that patients with ABMR had significantly lower TTV levels than those without rejection, and they demonstrated a linear dose–response relationship between TTV levels and alloreactivity, with approximately a 10% reduction in risk per log increase [56]. This finding has been further supported within the literature [57,58,59,60]. Additionally, the relationship between TTV and subclinical rejection was described by the Vienna group [61].

As patients are immunosuppressed there is an increased risk of infections. Often, patients are covered for opportunistic infections, but there is still a high incidence of morbidity and mortality from infections. Like rejection, TTV levels are associated with infection risk, with higher levels indicating greater immunosuppression and, consequently, an increased susceptibility to infection. This association was first highlighted by the TTV Quantification for the Prediction of Organ Rejection in Kidney Transplantation (TTV-POET trial-Vienna Group), in which higher TTV levels post-transplant was associated with increased infection risk, with an 11% rise in odds per log increase (OR 1.11; 95% CI 1.06–1.15; *p* < 0.001). This association was further supported once immunosuppression had stabilised, with significantly higher TTV levels observed at 1-month post-transplant in those who developed infections (4.6 vs. 3.8 log_10_ copies/mL; *p* = 0.023) or immunosuppression related adverse reactions (4.9 vs. 3.9 log_10_ copies/mL; *p* = 0.009), and with peak levels later predicting complications beyond 3 months [59]. Regarding cytomegalovirus viremia (CMV), one study demonstrated the TTV load within the first 10 days post-transplant correlated with higher CMV viremia [62].

BK viremia and nephropathy are often a consequence of overt immunosuppression. Therefore, the potential use of TTV as a surrogate marker to guide immunosuppression would be welcomed. Unfortunately, the relationship remains complex. In one study, no clear discriminatory TTV threshold differentiated patients with or without BK virus replication; however, TTV levels appeared indirectly related to BK virus risk depending on induction and maintenance regimens. For example, among antithymoglobulin-treated patients, those receiving cyclosporine had lower TTV levels (*p* < 0.01) and three-fold lower BK reactivation (13% vs. 37%) [63]. Another study found that, while baseline TTV levels were not predictive of BK viremia, higher TTV loads were associated with greater BK viremia over time; each 0.2 log_10_ rise in TTV load corresponded to a 1.5 log_10_ increase in BK load, and BK-viremic patients had persistently higher TTV at month 6 (6.93 vs. 5.47 log_10_ copies/mL; *p* = 0.015) [57]. Further research is needed to describe the relationship between TTV and BK viremia.

Overall, TTV levels inversely reflect immune competence and have emerged as a promising surrogate marker for immunosuppressive monitoring. Evidence from the Vienna group and others consistently shows that lower TTV levels are associated with heightened alloimmune activity, including both clinical and subclinical rejection, while higher levels correspond to increased risk of infection and immunosuppression-related complications. This dual association underscores TTV’s potential role in balancing immunosuppression to minimise both rejection and infection risk. Although thresholds may vary depending on immunosuppressive regimens and patient factors, TTV may offer a dynamic, non-invasive means of tailoring therapy.

### 4.3. Limitations of TTV

Despite the promise of TTV load as a non-invasive biomarker to guide immunosuppression, several limitations currently restrict its clinical utility.

Currently, no standardised interventional trial has confirmed that applying specific TTV thresholds improves clinical outcomes. While cut-offs for rejection (e.g., <4.6 log_10_ c/mL) and infection (e.g., >6.6 log_10_ c/mL) have been proposed based on observational data, these thresholds remain largely unvalidated in prospective randomised trials. Until such data are available, their application in routine care remains speculative [53].

Additionally, inter-assay variability limits broader adoption. TTV load quantification differs across platforms; for instance, the Vienna in-house PCR reports values ~1.4 log_10_ c/mL higher than the commercial R-GENE^®^ assay, while the Pisa assay underestimates values by ~0.3 log_10_ c/mL [17,53,64]. This lack of calibration limits cross-study comparability and universal threshold application.

Although biologically plausible and mechanistically appealing, the clinical implementation of TTV load as a biomarker requires further standardisation, assay harmonisation, and validation through well-designed prospective trials.

### 4.4. Future Directions of TTV

The European Union has funded the TTVguide project (EUCT: 2022-500024-30-00), which aims to investigate TTV as a tool to guide immunosuppression. The ongoing phase II trial conducted by the TTVguideIT group is applying the upper (>6.2 log10 c/mL) and lower (<4.6 log10 c/mL) thresholds, derived by calibrating in-house and commercial PCR assays, to guide tacrolimus dose. Additionally, the TTV-based Management Of Long-term Immunosuppression in Kidney Transplantation (TAOIST—NCT06829719) trial is investigating the application of TTV in guiding immunosuppression [64]. One single centre study already demonstrated the clinical application of TTV to guide immunosuppression [65]. However, larger multicentred randomised control trial data will provide granularity and dictate the future of TTV in kidney transplantation. If validated, TTV may serve as a practical, non-invasive tool to individualise immunosuppression and reduce the risk of both rejection and infection.

## 5. mRNA GEP

### 5.1. Background

Messenger RNA (mRNA) gene expression profiles (GEPs) in blood have also provided insight into rejection and have more specificity compared to dd-cfDNA. mRNA provides greater insight into biological activity and, with recent technological advances, has become a reliable and cost-effective biomarker in transplantation. Additionally, mRNA has the benefit of being amplified and is thus easily measurable compared to other proteins [66].

### 5.2. Clinical Utility

While prior CTOT studies (CTOT-1 and CTOT-4) explored prognostic gene expression profiles, CTOT-8 specifically validated a blood-based mRNA GEP for detecting subclinical acute rejection (subAR) [67]. At a predefined threshold, the test showed high specificity and a strong negative predictive value (NPV 78–88%) for ruling out subAR, while positive predictive value was moderate (47–61%). Importantly, the GEP and clinical phenotype independently correlated with adverse outcomes, including DSA, functional decline, and progression of chronic injury. The GEP also predicted lack of histologic improvement after subAR treatment, supporting its role in non-invasive monitoring and treatment response assessment.

To predict future graft loss, several studies have investigated the use of genomics measured at the time of routine biopsy. The Genomics of Chronic Allograft Rejection (GoCAR) study identified a 13-gene signature in early protocol biopsies (3 months post-transplant) that predicted fibrosis and allograft loss at one year [68]. This gene set outperformed conventional clinical and histological parameters, with an AUC of 0.967 for predicting future fibrosis and 0.84 for early graft loss, highlighting its potential to identify high-risk patients before irreversible injury occurs. In parallel, the European funded Biomarkers of Renal Graft Injuries (BIOMARGIN) study focused on the non-invasive detection of ABMR using peripheral blood [69]. The BIOMARGIN group derived and validated an 8-gene mRNA signature (including CXCL10, FCGR1A/B, GBP1/4, IL15, KLRC1, and TIMP1) that discriminated ABMR with an AUC of 79.9%. Notably, the diagnostic accuracy of the assay was maintained both early and late post-transplant, and in patients with stable function or graft dysfunction. The assay outperformed standard clinical indicators and showed added value in guiding decisions on whether to perform a biopsy.

Urinary mRNA has also shown promise as a non-invasive biomarker, although its use has been limited by the instability and rapid degradation of free mRNA in urine [70]. More recently, urinary exosomal mRNA has emerged as a robust alternative, protected within membrane-bound vesicles. A multicentre study demonstrated that a urinary exosome-derived mRNA signature could distinguish between rejection and no rejection with an AUC of 0.93, and between TCMR and ABMR with an AUC of 0.87. This outperformed traditional measures such as eGFR and proteinuria, with high negative and positive predictive values, thus supporting its utility for early detection [71].

Together, these studies support the clinical potential of peripheral blood, urine, and tissue-derived gene expression profiling in both early diagnosis of rejection phenotypes and prognostic enrichment.

### 5.3. Limitations of mRNA GEPs

Despite their promise, mRNA GEPs have several limitations. For example, blood-based mRNA signatures often require centralised processing and normalisation pipelines, limiting widespread clinical adoption. Standardisation across platforms and laboratories remains a challenge, particularly when comparing results across centres or integrating into multicentre trials [17].

Urinary biomarkers are promising as a non-invasive route. However, mRNA is limited by poor transcript stability due to enzymatic degradation in urine. This has limited the reproducibility and validation of urinary mRNA assays across cohorts. While amplification helps enhance detectability, it also introduces variability and requires careful quality control [17,70,72].

### 5.4. Future Directions of mRNA GEPs

Ongoing clinical trials, like the Clinical Utility of the Omnigraf biomarker panel In The Care of kidney Transplant Recipients (CLARITY) trial (NCT05482100), looks to prognosticate kidney transplant outcomes using the combination of a biomarker panel (OmniGraf^®^), which contains dd-cfDNA with mRNA [73]. Similarly, the GEnomic Medicine in Kidney Transplantation (GEM-KiT) study is investigating prognostic outcomes from genomic sequencing (NCT06365411). Therefore, these clinical trials may demonstrate the prognostic enrichment capabilities of mRNA GEPs, individually, or in combination with other biomarkers.

## 6. Discussion

Despite substantial advances in transplant immunology and short-term graft survival, long-term graft function remains a major clinical challenge using conventional diagnostic tools. There is a clear need for novel, non-invasive biomarkers that can enable earlier diagnosis and better long-term prognostication to support graft preservation [1,2,3]. This review illustrates that while several biomarkers show potential for earlier detection of allograft rejection, their clinical applications are context dependent. dd-cfDNA, for instance, is supported by commercial assays and multicentre validation but lacks specificity and may be elevated in non-rejection injury [33,34]. Urinary chemokines, such as CXCL9/10, offer promise for TCMR detection but are limited by confounders like infection. TTV reflects net immunosuppression and may help guide immunotherapy, although prospective interventional validation is lacking. mRNA GEPs offer exceptional diagnostic performance yet are constrained by technical and logistical barriers (Table 1).

The clinical utility of each biomarker is context-dependent and varies by post-transplant timepoint. Moving forward, it is increasingly evident that biomarkers will be most effective when integrated with traditional clinical tools such as serum creatinine, proteinuria, and DSA to guide clinical decisions [21,37]. dd-cfDNA appears most useful in the early post-transplant period for detecting acute rejection due to its high NPV, while urinary chemokines like CXCL9/10 may better reflect T-cell mediated inflammation during subclinical episodes [46]. TTV, by contrast, serves as a dynamic marker of immune suppression rather than rejection per se, and may be most relevant for guiding maintenance immunosuppression [56]. However, before broad implementation, further large-scale trials, standardisation of assay platforms, and validated thresholds are essential.

In this context, the specific strengths of each biomarker become evident. TTV may be best positioned as a screening tool to identify patients at risk of under-immunosuppression and subsequent rejection, offering a dynamic, longitudinal measure of immune competence [57,58,59,60]. In contrast, dd-cfDNA and urinary chemokines such as CXCL9/10 may serve as sensitive tools to rule out ongoing rejection episodes [14,15,22,46]. Meanwhile, mRNA GEPs offer greater specificity for active rejection and provide insight into chronic fibrotic processes that may be missed by dd-cfDNA [69]. These complementary roles support a layered, or panel, approach to biomarker-guided care, whereby different markers are leveraged according to their diagnostic strengths and clinical context.

Among the biomarkers reviewed, dd-cfDNA and mRNA GEPs are closest to routine implementation, given commercial availability and trial validation [17]. dd-cfDNA provides a dynamic measure of allograft injury but lacks specificity for rejection subtypes, whereas mRNA GEPs offer mechanistic insight into immune activation [31,66]. While each has limitations, their combined application, as being investigated in the CLARITY trial, may enhance diagnostic performance by improving sensitivity and specificity, thus enabling more accurate detection of rejection and assessment of immunosuppressive burden.

As urine is still an attractive non-invasive marker of kidney function, the use of CXCL and exosomal mRNA may provide effective screening tools for rejection [72]. However, the lack of specificity with CXCL9/10 with other renal pathologies, such as infection and acute kidney injury, highlight the need for multiple markers, such as exosomal mRNA, along with clinical contexts, to potentially guide clinical decisions. Future investigation of a panel of biomarkers, which may include the combination of the strong candidate biomarkers, may provide simultaneous insight into rejection, acute and subclinical, and guide immunosuppression.

Overall, the future of transplant rejection surveillance is likely to rely on a multi-biomarker strategy. While no single biomarker offers complete diagnostic certainty, their combined use may improve diagnostic and prognostic precision. As less-invasive biomarkers, they may provide guidance on biopsy timing and immunosuppression dosing and phenotypically enrich transplant rejection when used with Banff criteria and MMDx [6,13,14,15]. Moreover, as computational models and machine learning techniques evolve, there is potential to develop dynamic, personalised risk prediction tools based on biomarker trajectories, molecular diagnostics, and clinical context.

## 7. Conclusions

Despite advances in immunotherapy and insight into transplant rejection, outcomes remain a clinical challenge. Novel biomarkers are emerging as supportive diagnostic and prognostic markers within the field. Although each biomarker has its limitations, it is likely that a combination of biomarkers, the clinical question, and the patient’s risk factors and history will allow for personalised kidney management. Ultimately, while these biomarkers represent a leap forward in precision transplant medicine, their integration into routine practice will require further validation, harmonisation of assays, and demonstration of clinical utility in prospective, interventional trials. A major unmet need is identifying how these markers perform in combination and defining clinically actionable thresholds for their integrated use in practice.

## Figures and Tables

**Table 1:** Candidate biomarkers for kidney transplant rejection.

Biomarker	Use	Potential Thresholds and Accuracy	Limitations	Clinical Trials	References
Donor-Derived Cell-Free DNA (dd-cfDNA)	Detection of rejection Guide immunosuppression	Fraction: ≥1% Quantity: ≥75 cp/mL	Elevated in non-rejection injury (e.g., infection, trauma) Limited sensitivity for chronic changes (e.g., IFTA) Assay variability and confounding by recipient cfDNA	NCT04239703 NCT06013358 NCT06025240	Bloom et al. (2017) [20] Halloran et al. (2022; 2023) [14,15,22]
Urinary Chemokines (CXCL9/10)	Detection of ABMR and TCMR Risk stratification Predict response and progression	No universal cut-off AUCs 0.72–0.85	Non-specific elevation in infection, AKI, and autoimmune disease Requires context-aware interpretation	NCT03206801 NCT06564649 NCT06351488 NCT03719339	Tinel et al. (2020) [37] Van Loon et al. (2024) [46]
Torque Teno Virus (TTV)	Surrogate marker of immune function Predict infection and rejection risk Tailor immunosuppression	<4.6 log_10_ c/mL → higher rejection risk >6.6 log_10_ c/mL → higher infection risk	No validated thresholds from RCTs Inter-assay variability (e.g., Vienna vs. commercial assays) Influence of induction and maintenance immunosuppression regimens	EUCT: 2022-500024-30-00 NCT06829719	Schiemann et al. (2017) [55] Strassl et al. (2019) [56] Doberer et al. (2020) [60]
mRNA Gene Expression Profiles (GEPs)	Detect subclinical rejection Prognosticate fibrosis and graft loss Treatment response	Study-specific signatures AUCs up to 0.97	Requires centralised processing Lack of assay standardisation Urinary mRNA limited by transcript degradation; improved with exosomal mRNA	NCT05482100 NCT06365411	Suthanthiran et al. (2013) [66] O’Connel et al. (2016) [68] Vanhove et al. (2022) [69]

IFTA—Interstitial Fibrosis and Tubular Atrophy. ABMR—Anti-body mediated rejection. TCMR—T-Cell mediate Rejection. AKI—Acute kidney injury. AUC—Area under the receiver operating characteristic curve.

## Data Availability

No new data were created or analyzed in this study.

## References

[1] Oellerich M., Sherwood K., Keown P., Schutz E., Beck J., Stegbauer J., Rump L.C., Walson P.D. (2021). Liquid biopsies: Donor-derived cell-free DNA for the detection of kidney allograft injury. Nat. Rev. Nephrol..

[2] Hariharan S., Israni A.K., Danovitch G. (2021). Long-Term Survival after Kidney Transplantation. N. Engl. J. Med..

[3] Hart A., Lentine K.L., Smith J.M., Miller J.M., Skeans M.A., Prentice M., Robinson A., Foutz J., Booker S.E., Israni A.K. (2021). OPTN/SRTR 2019 Annual Data Report: Kidney. Am. J. Transplant..

[4] Tamargo C.L., Kant S. (2023). Pathophysiology of Rejection in Kidney Transplantation. J. Clin. Med..

[5] Anglicheau D., Naesens M., Essig M., Gwinner W., Marquet P. (2016). Establishing Biomarkers in Transplant Medicine: A Critical Review of Current Approaches. Transplantation.

[6] Haas M., Loupy A., Lefaucheur C., Roufosse C., Glotz D., Seron D., Nankivell B.J., Halloran P.F., Colvin R.B., Akalin E. (2018). The Banff 2017 Kidney Meeting Report: Revised diagnostic criteria for chronic active T cell-mediated rejection, antibody-mediated rejection, and prospects for integrative endpoints for next-generation clinical trials. Am. J. Transplant..

[7] Jeong H.J. (2020). Diagnosis of renal transplant rejection: Banff classification and beyond. Kidney Res. Clin. Pract..

[8] Solez K., Colvin R.B., Racusen L.C., Haas M., Sis B., Mengel M., Halloran P.F., Baldwin W., Banfi G., Collins A.B. (2008). Banff 07 classification of renal allograft pathology: Updates and future directions. Am. J. Transplant..

[9] Schinstock C.A., Sapir-Pichhadze R., Naesens M., Batal I., Bagnasco S., Bow L., Campbell P., Clahsen-van Groningen M.C., Cooper M., Cozzi E. (2019). Banff survey on antibody-mediated rejection clinical practices in kidney transplantation: Diagnostic misinterpretation has potential therapeutic implications. Am. J. Transplant..

[10] Halloran P.F., Famulski K.S., Reeve J. (2016). Molecular assessment of disease states in kidney transplant biopsy samples. Nat. Rev. Nephrol..

[11] Einecke G., Sis B., Reeve J., Mengel M., Campbell P.M., Hidalgo L.G., Kaplan B., Halloran P.F. (2009). Antibody-mediated microcirculation injury is the major cause of late kidney transplant failure. Am. J. Transplant..

[12] Loupy A., Lefaucheur C., Vernerey D., Chang J., Hidalgo L.G., Beuscart T., Verine J., Aubert O., Dubleumortier S., Duong van Huyen J.P. (2014). Molecular microscope strategy to improve risk stratification in early antibody-mediated kidney allograft rejection. J. Am. Soc. Nephrol..

[13] Gupta G., Moinuddin I., Kamal L., King A.L., Winstead R., Demehin M., Kang L., Kimball P., Levy M., Bhati C. (2022). Correlation of Donor-derived Cell-free DNA With Histology and Molecular Diagnoses of Kidney Transplant Biopsies. Transplantation.

[14] Halloran P.F., Reeve J., Madill-Thomsen K.S., Demko Z., Prewett A., Billings P., Trifecta I. (2022). The Trifecta Study: Comparing Plasma Levels of Donor-derived Cell-Free DNA with the Molecular Phenotype of Kidney Transplant Biopsies. J. Am. Soc. Nephrol..

[15] Halloran P.F., Reeve J., Madill-Thomsen K.S., Kaur N., Ahmed E., Cantos C., Al Haj Baddar N., Demko Z., Liang N., Swenerton R.K. (2022). Combining Donor-derived Cell-free DNA Fraction and Quantity to Detect Kidney Transplant Rejection Using Molecular Diagnoses and Histology as Confirmation. Transplantation.

[16] Weidmann L., Harmacek D., Gaspert A., Helmchen B.M., George B., Hübel K., von Moos S., Rho E., Schachtner T. (2025). Biopsy-Based Transcriptomics Support Rejection Monitoring Through Repeated Kidney Allograft Biopsies. Kidney Int. Rep..

[17] Westphal S.G., Mannon R.B. (2025). Biomarkers of Rejection in Kidney Transplantation. Am. J. Kidney Dis..

[18] Beck J., Bierau S., Balzer S., Andag R., Kanzow P., Schmitz J., Gaedcke J., Moerer O., Slotta J.E., Walson P. (2013). Digital droplet PCR for rapid quantification of donor DNA in the circulation of transplant recipients as a potential universal biomarker of graft injury. Clin. Chem..

[19] Bromberg J.S., Brennan D.C., Poggio E., Bunnapradist S., Langone A., Sood P., Matas A.J., Mannon R.B., Mehta S., Sharfuddin A. (2017). Biological Variation of Donor-Derived Cell-Free DNA in Renal Transplant Recipients: Clinical Implications. J. Appl. Lab. Med..

[20] Bloom R.D., Bromberg J.S., Poggio E.D., Bunnapradist S., Langone A.J., Sood P., Matas A.J., Mehta S., Mannon R.B., Sharfuddin A. (2017). Cell-Free DNA and Active Rejection in Kidney Allografts. J. Am. Soc. Nephrol..

[21] Aubert O., Ursule-Dufait C., Brousse R., Gueguen J., Racape M., Raynaud M., Van Loon E., Pagliazzi A., Huang E., Jordan S.C. (2024). Cell-free DNA for the detection of kidney allograft rejection. Nat. Med..

[22] Halloran P.F., Reeve J., Madill-Thomsen K.S., Demko Z., Prewett A., Gauthier P., Billings P., Lawrence C., Lowe D., Hidalgo L.G. (2023). Antibody-mediated Rejection Without Detectable Donor-specific Antibody Releases Donor-derived Cell-free DNA: Results From the Trifecta Study. Transplantation.

[23] Bunnapradist S., Homkrailas P., Ahmed E., Fehringer G., Billings P.R., Tabriziani H. (2021). Using both the Fraction and Quantity of Donor-Derived Cell-Free DNA to Detect Kidney Allograft Rejection. J. Am. Soc. Nephrol..

[24] Wijtvliet V.P., Plaeke P., Abrams S., Hens N., Gielis E.M., Hellemans R., Massart A., Hesselink D.A., De Winter B.Y., Abramowicz D. (2020). Donor-derived cell-free DNA as a biomarker for rejection after kidney transplantation: A systematic review and meta-analysis. Transpl. Int..

[25] Knight S.R., Thorne A., Lo Faro M.L. (2019). Donor-specific Cell-free DNA as a Biomarker in Solid Organ Transplantation. A Systematic Review. Transplantation.

[26] Xing Y., Guo Q., Wang C., Shi H., Zheng J., Jia Y., Li C., Hao C. (2024). Donor-derived cell-free DNA as a diagnostic marker for kidney-allograft rejection: A systematic review and meta-analysis. Biomol. Biomed..

[27] Sawinski D.L., Mehta S., Alhamad T., Bromberg J.S., Fischbach B., Aeschbacher T., Ghosh S., Shekhtman G., Dholakia S., Brennan D.C. (2021). Association between dd-cfDNA levels, de novo donor specific antibodies, and eGFR decline: An analysis of the DART cohort. Clin. Transplant..

[28] Bu L., Gupta G., Pai A., Anand S., Stites E., Moinuddin I., Bowers V., Jain P., Axelrod D.A., Weir M.R. (2022). Clinical outcomes from the Assessing Donor-derived cell-free DNA Monitoring Insights of kidney Allografts with Longitudinal surveillance (ADMIRAL) study. Kidney Int..

[29] Shen J., Guo L., Yan P., Zhou J., Zhou Q., Lei W., Liu H., Liu G., Lv J., Liu F. (2020). Prognostic value of the donor-derived cell-free DNA assay in acute renal rejection therapy: A prospective cohort study. Clin. Transplant..

[30] Wolf-Doty T.K., Mannon R.B., Poggio E.D., Hinojosa R.J., Hiller D., Bromberg J.S., Brennan D.C. (2021). Dynamic Response of Donor-Derived Cell-Free DNA Following Treatment of Acute Rejection in Kidney Allografts. Kidney360.

[31] Kant S., Bromberg J., Haas M., Brennan D. (2020). Donor-derived Cell-free DNA and the Prediction of BK Virus-associated Nephropathy. Transpl. Direct.

[32] Gielis E.M., Beirnaert C., Dendooven A., Meysman P., Laukens K., De Schrijver J., Van Laecke S., Van Biesen W., Emonds M.P., De Winter B.Y. (2018). Plasma donor-derived cell-free DNA kinetics after kidney transplantation using a single tube multiplex PCR assay. PLoS ONE.

[33] Schutz E., Asendorf T., Beck J., Schauerte V., Mettenmeyer N., Shipkova M., Wieland E., Kabakchiev M., Walson P.D., Schwenger V. (2020). Time-Dependent Apparent Increase in dd-cfDNA Percentage in Clinically Stable Patients Between One and Five Years Following Kidney Transplantation. Clin. Chem..

[34] Oellerich M., Shipkova M., Asendorf T., Walson P.D., Schauerte V., Mettenmeyer N., Kabakchiev M., Hasche G., Grone H.J., Friede T. (2019). Absolute quantification of donor-derived cell-free DNA as a marker of rejection and graft injury in kidney transplantation: Results from a prospective observational study. Am. J. Transplant..

[35] Huang E., Mengel M., Clahsen-van Groningen M.C., Jackson A.M. (2023). Diagnostic Potential of Minimally Invasive Biomarkers: A Biopsy-centered Viewpoint From the Banff Minimally Invasive Diagnostics Working Group. Transplantation.

[36] Bromberg J.S., Bunnapradist S., Samaniego-Picota M., Anand S., Stites E., Gauthier P., Demko Z., Prewett A., Armer-Cabral M., Marshall K. (2024). Elevation of Donor-derived Cell-free DNA Before Biopsy-proven Rejection in Kidney Transplant. Transplantation.

[37] Tinel C., Devresse A., Vermorel A., Sauvaget V., Marx D., Avettand-Fenoel V., Amrouche L., Timsit M.O., Snanoudj R., Caillard S. (2020). Development and validation of an optimized integrative model using urinary chemokines for noninvasive diagnosis of acute allograft rejection. Am. J. Transplant..

[38] Hu H., Kwun J., Aizenstein B.D., Knechtle S.J. (2009). Noninvasive detection of acute and chronic injuries in human renal transplant by elevation of multiple cytokines/chemokines in urine. Transplantation.

[39] Ho J., Schaub S., Jackson A.M., Balshaw R., Carroll R., Cun S., De Serres S.A., Fantus D., Handschin J., Honger G. (2023). Multicenter Validation of a Urine CXCL10 Assay for Noninvasive Monitoring of Renal Transplants. Transplantation.

[40] Gao J., Wu L., Wang S., Chen X. (2020). Role of Chemokine (C-X-C Motif) Ligand 10 (CXCL10) in Renal Diseases. Mediat. Inflamm..

[41] Hricik D.E., Nickerson P., Formica R.N., Poggio E.D., Rush D., Newell K.A., Goebel J., Gibson I.W., Fairchild R.L., Riggs M. (2013). Multicenter validation of urinary CXCL9 as a risk-stratifying biomarker for kidney transplant injury. Am. J. Transplant..

[42] Janfeshan S., Afshari A., Yaghobi R., Roozbeh J. (2024). Urinary CXCL-10, a prognostic biomarker for kidney graft injuries: A systematic review and meta-analysis. BMC Nephrol..

[43] Jackson J.A., Kim E.J., Begley B., Cheeseman J., Harden T., Perez S.D., Thomas S., Warshaw B., Kirk A.D. (2011). Urinary chemokines CXCL9 and CXCL10 are noninvasive markers of renal allograft rejection and BK viral infection. Am. J. Transplant..

[44] Rabant M., Amrouche L., Morin L., Bonifay R., Lebreton X., Aouni L., Benon A., Sauvaget V., Le Vaillant L., Aulagnon F. (2016). Early Low Urinary CXCL9 and CXCL10 Might Predict Immunological Quiescence in Clinically and Histologically Stable Kidney Recipients. Am. J. Transplant..

[45] Suthanthiran M., Schwartz J.E., Ding R., Abecassis M., Dadhania D., Samstein B., Knechtle S.J., Friedewald J., Becker Y.T., Sharma V.K. (2013). Urinary-cell mRNA profile and acute cellular rejection in kidney allografts. N. Engl. J. Med..

[46] Van Loon E., Tinel C., de Loor H., Bossuyt X., Callemeyn J., Coemans M., De Vusser K., Sauvaget V., Olivre J., Koshy P. (2024). Automated Urinary Chemokine Assays for Noninvasive Detection of Kidney Transplant Rejection: A Prospective Cohort Study. Am. J. Kidney Dis..

[47] Goutaudier V., Aubert O., Racapé M., Truchot A., Sablik M., Raynaud M., Vicaut É., Rousseau O., Elias M., Divard G. (2024). Detection of Kidney Allograft Rejection Using Urinary Chemokines. J. Am. Soc. Nephrol..

[48] Vaidya V.S., Waikar S.S., Ferguson M.A., Collings F.B., Sunderland K., Gioules C., Bradwin G., Matsouaka R., Betensky R.A., Curhan G.C. (2008). Urinary biomarkers for sensitive and specific detection of acute kidney injury in humans. Clin. Transl. Sci..

[49] Canney M., Clark E.G., Hiremath S. (2023). Biomarkers in acute kidney injury: On the cusp of a new era?. J. Clin. Investig..

[50] Rocchi J., Ricci V., Albani M., Lanini L., Andreoli E., Macera L., Pistello M., Ceccherini-Nelli L., Bendinelli M., Maggi F. (2009). Torquetenovirus DNA drives proinflammatory cytokines production and secretion by immune cells via toll-like receptor 9. Virology.

[51] Chen T., Väisänen E., Mattila P.S., Hedman K., Söderlund-Venermo M. (2013). Antigenic diversity and seroprevalences of Torque teno viruses in children and adults by ORF2-based immunoassays. J. Gen. Virol..

[52] Focosi D., Antonelli G., Pistello M., Maggi F. (2016). Torquetenovirus: The human virome from bench to bedside. Clin. Microbiol. Infect..

[53] Jaksch P., Gorzer I., Puchhammer-Stockl E., Bond G. (2022). Integrated Immunologic Monitoring in Solid Organ Transplantation: The Road Toward Torque Teno Virus-guided Immunosuppression. Transplantation.

[54] Zeng J., Tang Y., Lin T., Song T. (2023). Torque-teno virus for the prediction of graft rejection and infection disease after kidney transplantation: A systematic review and meta-analysis. J. Med. Virol..

[55] Schiemann M., Puchhammer-Stockl E., Eskandary F., Kohlbeck P., Rasoul-Rockenschaub S., Heilos A., Kozakowski N., Gorzer I., Kikic Z., Herkner H. (2017). Torque Teno Virus Load-Inverse Association With Antibody-Mediated Rejection After Kidney Transplantation. Transplantation.

[56] Strassl R., Doberer K., Rasoul-Rockenschaub S., Herkner H., Gorzer I., Klager J.P., Schmidt R., Haslacher H., Schiemann M., Eskandary F.A. (2019). Torque Teno Virus for Risk Stratification of Acute Biopsy-Proven Alloreactivity in Kidney Transplant Recipients. J. Infect. Dis..

[57] Solis M., Velay A., Gantner P., Bausson J., Filipputtu A., Freitag R., Moulin B., Caillard S., Fafi-Kremer S. (2019). Torquetenovirus viremia for early prediction of graft rejection after kidney transplantation. J. Infect..

[58] van Rijn A.L., Wunderink H.F., Sidorov I.A., de Brouwer C.S., Kroes A.C., Putter H., de Vries A.P., Rotmans J.I., Feltkamp M.C. (2021). Torque teno virus loads after kidney transplantation predict allograft rejection but not viral infection. J. Clin. Virol..

[59] Fernández-Ruiz M., Albert E., Giménez E., Ruiz-Merlo T., Parra P., López-Medrano F., San Juan R., Polanco N., Andrés A., Navarro D. (2019). Monitoring of alphatorquevirus DNA levels for the prediction of immunosuppression-related complications after kidney transplantation. Am. J. Transplant..

[60] Doberer K., Schiemann M., Strassl R., Haupenthal F., Dermuth F., Görzer I., Eskandary F., Reindl-Schwaighofer R., Kikić Ž., Puchhammer-Stöckl E. (2020). Torque teno virus for risk stratification of graft rejection and infection in kidney transplant recipients—A prospective observational trial. Am. J. Transplant..

[61] Doberer K., Haupenthal F., Nackenhorst M., Bauernfeind F., Dermuth F., Eigenschink M., Schiemann M., Kläger J., Görzer I., Eskandary F. (2021). Torque teno virus load is associated with subclinical alloreactivity in kidney transplant recipients: A prospective observational trial. Transplantation.

[62] Maggi F., Focosi D., Statzu M., Bianco G., Costa C., Macera L., Spezia P.G., Medici C., Albert E., Navarro D. (2018). Early Post-Transplant Torquetenovirus Viremia Predicts Cytomegalovirus Reactivations In Solid Organ Transplant Recipients. Sci. Rep..

[63] Handala L., Descamps V., Morel V., Castelain S., Francois C., Duverlie G., Helle F., Brochot E. (2019). No correlation between Torque Teno virus viral load and BK virus replication after kidney transplantation. J. Clin. Virol..

[64] CORDIS Personalisation of Immunosuppression by Monitoring Viral Load Post Kidney Transplantation—A Randomised Controlled Phase II Trial. https://cordis.europa.eu/project/id/896932.

[65] Reineke M., Speer C., Bundschuh C., Klein J.A.F., Loi L., Sommerer C., Zeier M., Schnitzler P., Morath C., Benning L. (2024). Impact of induction agents and maintenance immunosuppression on torque teno virus loads and year-one complications after kidney transplantation. Front. Immunol..

[66] Halloran P.F., Venner J.M., Madill-Thomsen K.S., Einecke G., Parkes M.D., Hidalgo L.G., Famulski K.S. (2018). Review: The transcripts associated with organ allograft rejection. Am. J. Transplant..

[67] Friedewald J.J., Kurian S.M., Heilman R.L., Whisenant T.C., Poggio E.D., Marsh C., Baliga P., Odim J., Brown M.M., Ikle D.N. (2019). Development and clinical validity of a novel blood-based molecular biomarker for subclinical acute rejection following kidney transplant. Am. J. Transplant..

[68] O'Connell P.J., Zhang W., Menon M.C., Yi Z., Schroppel B., Gallon L., Luan Y., Rosales I.A., Ge Y., Losic B. (2016). Biopsy transcriptome expression profiling to identify kidney transplants at risk of chronic injury: A multicentre, prospective study. Lancet.

[69] Van Loon E., Gazut S., Yazdani S., Lerut E., de Loor H., Coemans M., Noel L.H., Thorrez L., Van Lommel L., Schuit F. (2019). Development and validation of a peripheral blood mRNA assay for the assessment of antibody-mediated kidney allograft rejection: A multicentre, prospective study. EBioMedicine.

[70] Ramalhete L., Araujo R., Ferreira A., Calado C.R.C. (2024). Exosomes and microvesicles in kidney transplantation: The long road from trash to gold. Pathology.

[71] El Fekih R., Hurley J., Tadigotla V., Alghamdi A., Srivastava A., Coticchia C., Choi J., Allos H., Yatim K., Alhaddad J. (2021). Discovery and Validation of a Urinary Exosome mRNA Signature for the Diagnosis of Human Kidney Transplant Rejection. J. Am. Soc. Nephrol..

[72] Lubetzky M.L., Salinas T., Schwartz J.E., Suthanthiran M. (2021). Urinary Cell mRNA Profiles Predictive of Human Kidney Allograft Status. Clin. J. Am. Soc. Nephrol..

[73] Fleming J.N., Cober T., Hickey J., Stach L., Kawano A., Szczepanik A., Watson A., Imamura Y., Weems J., West-Thielke P. (2022). Clinical Utility of the OmniGraf Biomarker Panel in the Care of Kidney Transplant Recipients (CLARITY): Protocol for a Prospective, Multisite Observational Study. JMIR Res. Protoc..

